# Field evaluation of the establishment potential of *w*melpop *Wolbachia* in Australia and Vietnam for dengue control

**DOI:** 10.1186/s13071-015-1174-x

**Published:** 2015-10-28

**Authors:** Tran Hien Nguyen, H. Le Nguyen, Thu Yen Nguyen, Sinh Nam Vu, Nhu Duong Tran, T. N. Le, Quang Mai Vien, T. C. Bui, Huu Tho Le, Simon Kutcher, Tim P. Hurst, T. T. H. Duong, Jason A. L. Jeffery, Jonathan M. Darbro, B. H. Kay, Iñaki Iturbe-Ormaetxe, Jean Popovici, Brian L. Montgomery, Andrew P. Turley, Flora Zigterman, Helen Cook, Peter E. Cook, Petrina H. Johnson, Peter A. Ryan, Chris J. Paton, Scott A. Ritchie, Cameron P. Simmons, Scott L. O’Neill, Ary A. Hoffmann

**Affiliations:** National Institute of Hygiene and Epidemiology, Hanoi, Viet Nam; Institute Pasteur, Nha Trang, Viet Nam; Khanh Hoa Health Department, Nha Trang, Viet Nam; Australian Foundation for Peoples of Asia and the Pacific Limited, Hanoi, Viet Nam; QIMR Berghofer Medical Research Institute, Herston, Australia; School of Biological Sciences, Monash University, Melbourne, Australia; School of Public Health, Tropical Medicine and Rehabilitation Sciences, James Cook University, Smithfield, Australia; Oxford University Clinical Research Unit, Hospital for Tropical Diseases, Ho Chi Minh City, Viet Nam; Centre for Tropical Medicine, University of Oxford, Churchill Hospital, Oxford, UK; Department of Microbiology and Immunology, University of Melbourne, Parkville, Australia; Bio21 Institute and School of BioSciences, University of Melbourne, Parkville, Australia

**Keywords:** *Wolbachia*, Dengue, Release, *Aedes*, Fitness, Invasion

## Abstract

**Background:**

Introduced *Wolbachia* bacteria can influence the susceptibility of *Aedes aegypti* mosquitoes to arboviral infections as well as having detrimental effects on host fitness. Previous field trials demonstrated that the *w*Mel strain of *Wolbachia* effectively and durably invades *Ae. aegypti* populations. Here we report on trials of a second strain, *w*MelPop-PGYP *Wolbachia,* in field sites in northern Australia (Machans Beach and Babinda) and central Vietnam (Tri Nguyen, Hon Mieu Island), each with contrasting natural *Ae. aegypti* densities.

**Methods:**

Mosquitoes were released at the adult or pupal stages for different lengths of time at the sites depending on changes in *Wolbachia* frequency as assessed through PCR assays of material collected through Biogents-Sentinel (BG-S) traps and ovitraps. Adult numbers were also monitored through BG-S traps. Changes in *Wolbachia* frequency were compared across hamlets or house blocks.

**Results:**

Releases of adult *w*MelPop*-Ae. aegypti* resulted in the transient invasion of *w*MelPop in all three field sites. Invasion at the Australian sites was heterogeneous, reflecting a slower rate of invasion in locations where background mosquito numbers were high. In contrast, invasion across Tri Nguyen was relatively uniform. After cessation of releases, the frequency of *w*MelPop declined in all sites, most rapidly in Babinda and Tri Nguyen. Within Machans Beach the rate of decrease varied among areas, and *w*MelPop was detected for several months in an area with a relatively low mosquito density.

**Conclusions:**

These findings highlight challenges associated with releasing *Wolbachia*-*Ae. aegypti* combinations with low fitness, albeit strong virus interference properties, as a means of sustainable control of dengue virus transmission.

**Electronic supplementary material:**

The online version of this article (doi:10.1186/s13071-015-1174-x) contains supplementary material, which is available to authorized users.

## Background

Dengue is the most prevalent arboviral disease of humans, with an estimated 390 million cases per year including 96 million clinical cases [1]. As there is no commercially available vaccine, control of the primary mosquito vector, *Aedes aegypti,* has long been the backbone of public health efforts to reduce dengue transmission, yet this has not been sustainable in any dengue endemic country. *Aedes aegypti* stably infected with *Wolbachia* endosymbionts are less susceptible to infection with medically important arboviruses, including dengue viruses (DENV) [2–5]. This factor and other impacts of *Wolbachia* on the life history of *Ae. aegypti* [6–9] has generated interest in establishing *Wolbachia* infections in mosquito populations as a means of controlling transmission of DENV.

In 2011 the *Wolbachia* strain *w*Mel was successfully introduced into *Ae. aegypti* populations in northern Australian field sites [10] where it has now remained at a frequency >90 % for more than 3 years [11, 12]. The *w*Mel strain reduces the susceptibility of *Ae. aegypti* to infection by multiple serotypes of dengue [5, 11]. While this level of reduced susceptibility is projected to be sufficient to substantially lower the incidence of dengue in human populations [13], the availability of alternative strains e.g., *w*MelPop-PGYP (hereafter referred to as *w*MelPop) with even greater resistance to DENV infection [3, 13, 14] also warrants their field testing.

The *w*MelPop-*Ae. aegypti* combination was generated with the aim to reduce lifespan and thereby interfere with virus transmission because reductions in mosquito lifespan are known to have significant impacts on vectorial capacity of mosquitoes [6]. Subsequently, it was shown that *w*MelPop very effectively reduced replication of arboviruses in *Ae. aegypti* [3, 14]. However *w*MelPop has physiological effects on hosts, including neurological and reproductive perturbations that can result in alterations to lifespan, egg development and hatching, egg quiescence, host probing and feeding, salivation, and larval development [7, 15–19]. Although *w*MelPop has successfully invaded field cages, albeit at a slower rate than *w*Mel [5], the deleterious host effects mean that successful establishment will depend on the infection exceeding a relatively high unstable equilibrium frequency. This unstable point has been estimated at around 40 % in the wet season, but is likely to be much higher in the dry season because the infection has a large impact on egg hatch rates when the eggs are dried and maintained in a quiescent state between flooding events [7, 19]. In the dry season, the unstable point may be as high as 80 or even 90 % [19] making establishment unlikely – and spatial spread from localized releases impossible [20, 21]. However the infection may result in suppression of the mosquito population if it is present at a high frequency [7, 8].

**Fig. 1:**
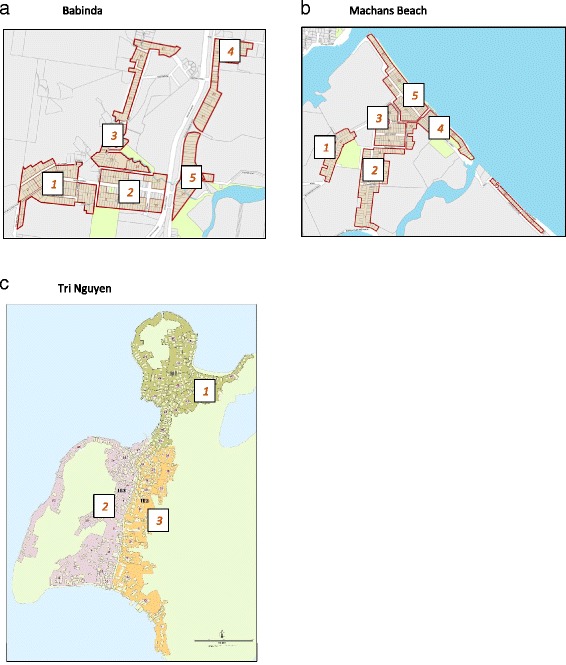
Map of (**a**) Babinda, (**b**) Machans Beach and (**c**) Tri Nguyen where releases were undertaken. Numbers indicate main blocks comprising the release area

In the current set of open field releases, we tested if *w*MelPop could be stably introduced into *Ae. aegypti* populations in three relatively isolated field sites (Fig. 1) in northern Australia (Machans Beach and Babinda), and central Vietnam (Tri Nguyen, Hon Mieu Island), where dengue is endemic. The findings suggest *w*MelPop releases might result in a high level of infected individuals during and after the release period which might lead to reductions of dengue transmission [13]. However the results also indicate that the release of *w*MelPop mosquitoes would need to be ongoing. This does not represent a self-sustaining intervention as would be the case for other *Wolbachia* strains like *w*Mel that have been shown to successfully persist after a relatively small introduction of infected mosquitoes [12].

## Methods

### Ethics statement

The release of *Aedes aegypti* containing *Wolbachia* was approved by the Australian Pesticides and Veterinary Medicines Authority (permit numbers 13183 and 13718). The release was regulated under existing legislation as a Veterinary Chemical product. For rearing mosquitoes requiring human bloodfeeding for Australian releases, Human Ethics Approval H4907 was provided by Human Research Ethics Committee, James Cook University (Human Ethics Advisor: Julie Parison; Head of Committee: Anne Swinbourne). All adult subjects provided informed oral consent (no children were involved). Names of subjects providing oral consents were recorded in writing. Written consents were not taken because this was not required by the ethics committee.

In Vietnam, the release of *Aedes aegypti* carrying *w*MelPop *Wolbachia* at Tri Nguyen, along with human blood feeding for colony maintenance, was approved by the internal review board (IRB) of the National Institute of Hygiene and Epidemiology (Approval reference number: 32IRB) and then the IRB of Vietnam Ministry of Health (Approval reference number: 615/CN-BYT). All adult subjects provided informed oral consent (no children were involved). Names of subjects providing oral consents were recorded in writing. Written consents were not taken because this was not required by the ethics committee.

### Releases in Australia

Releases occurred in Machans Beach (MB) and Babinda (BA). MB is 10 km north of Cairns while BA is 60 km south of Cairns (Fig. 1). MB consists of around 430 residences and covers an area of 0.47 km^2^; MB has large populations of *Ae. aegypti* and a history of dengue outbreaks [22, 23]. BA has 390 residences and covers 1.18 km^2^.

Adults for releases were reared following the procedure outlined previously [10]. Cairns strain *Ae. aegypti* infected with *w*MelPop-PGYP were reared in semi-field cages as described by Hoffmann *et al.* [10]. Briefly, larvae (500/bucket) were reared in 3 L buckets and fed a diet of Tetramin tropical fish flakes. When approximately 90 % of larvae had pupated, 50–100 larvae/pupae were placed into 750 ml plastic cups. Adult mosquitoes emerged over the following 3–5 days and were allowed access to a carbohydrate source before release. About 90 % of reared adults were used for release, with the remaining 10 % used to restock the colony. Stock adult mosquitoes were allowed to emerge in the semi-field cage, and females were blood fed on human volunteers (JCU ethics approval H3555) 5 times per week. Quality of the release material was measured weekly by taking a random sample of larvae and adult females that were tested for *w*MelPop infection by PCR [24]. The *w*MelPop colony had originally been backcrossed for five generations to wild type uninfected material from Cairns to ensure that the nuclear genetic background was >90 % that of the target population. Prior to release and while building up the population, it was then crossed weekly for another month with Cairns wild strain (F1) by introducing recently emerged wild males at a ratio of 10 % into the cage. After a month of backcrossing with wild *Ae. aegypti* it was found that the colony was losing *w*MelPop infection; to maintain infection the decision was made to cease backcrossing.

Releases were initiated on 4 January 2012 during the warm wet season (Additional file 1: Figure S1) and consisted of adults released at every 4^th^ house following the protocol outlined elsewhere [10]. The total number of released adults varied from between 13 and 28 females per house for BA, and between 13 and 27 females per house for MB. For BA 15 releases took place across a period of 4 months involving a similar number of males and females. For MB the initial release period followed that of BA, but after a gap of around 6 weeks another second release cycle was initiated in the dry season (Additional file 1: Figure S1) consisting of between 1700 and 6500 males per week and a smaller number of females (between 21 and 2200). The male bias was generated by sourcing adults from the mosquitoes eclosing on the first day of emergence in the rearing containers. The male releases were aimed at testing if *w*MelPop invasion might be facilitated by introducing males into a population expected to generate cytoplasmic incompatibility when mated with emerging uninfected females that would reduce the resident uninfected population.

### Monitoring *Wolbachia* frequencies in Australia

For the purposes of the releases and monitoring, MB was initially divided into 21 blocks, while BA was divided into 24 blocks (Fig. 1). These blocks were further combined into 5 (BA) or 6 (MB) larger blocks for monitoring (see below) with different residence characteristics within these blocks. To monitor the size of the mosquito populations and proportion with *w*MelPop infection, a network of 12 BG-S traps was established at BA and 10 at MB starting from October 2011. These were cleared weekly and mosquitoes sorted by species and sex. As well as obtaining data from the BG –S traps located in BA and MB on *Wolbachia* frequencies, we also used numbers in these traps to consider population changes across time after releases were completed. We assessed changes in *Ae. aegypti* numbers.

Initial monitoring of *Wolbachia* frequencies involved ovitraps, following the approach taken in Hoffmann et al. [10]. For this purpose, around 70 plastic buckets were placed out in backyards at each location. These buckets sampled *Wolbachia* frequencies in larvae (i.e., offspring of adults that had successfully fed and oviposited) with a maximum of 10 larvae sampled per container (although far fewer larvae were sampled from most containers). Between January and July, there were 6 ovitrap surveys at BA and 12 at MB. From 19 April 2012, the *Wolbachia* frequency was also monitored through an expanded network of 20 BG-Sentinel traps [25] placed throughout each area and collected weekly at both sites. In preliminary screening it was found that adults could be reliably scored for *Wolbachia* even if they had been in BG traps for a week. MelPop *Wolbachia* was detected in larval or adult samples by real time Taqman PCR using methods described previously [10], with exception that a primer/probe combination specific for the *w*MelPop-CLA strain was used [24]. The detection of *Wolbachia* in larvae was identical to the detection in adults, except that larva were not homogenised with glass beads prior to the DNA extraction step at 56 °C. Note that both types of traps collect adults and their offspring from both released mosquitoes and those of the natural population. Infection frequencies during the release period are therefore inflated.

### Colonies, maintenance and QA in Vietnam (Wolbachia and screening for CHIK/DENV)

The *w*MelPop colony was backcrossed for seven generations by mating virgin females to uninfected males from Tri Nguyen (TNI). While building up the populations for releases, the colony was then crossed weekly with further material from the TNI wild strain (F1) by introducing recently emerged wild males at a ratio of 10 % into the cage for 7 generations. Two colonies (release stock and back-up) were maintained in two insectaries located at the National Institute of Hygiene and Epidemiology (NIHE), Hanoi, Vietnam. Both colonies had 30 cages, stocked at a density of around 300 females, and blood fed weekly. Volunteer blood feeders were excluded if their temperature was 38 °C or above, if they had been taking antibiotics in the last 5 days or if they had been experiencing dengue like symptoms. Each week, eggs were collected from containers lined with filter paper, each cage producing approximately 6,000 eggs. The release stock colony and backup colonies produced a minimum of 180000 eggs per week for delivery (via courier and airplane) to the Institute Pasteur Nha Trang (INPT). Four to five days after bloodfeeding, 10 adult mosquitoes were sampled from the cages and screened for DENV to ensure that release colony mosquitoes were not exposed to DENV during the mass rearing process.

### Releases in Vietnam

Releases occurred at Tri Nguyen village, located on Hon Mieu island, Khanh Hoa province, central Vietnam. The island lies approximately 1 km from Nha Trang city on the mainland, and is approximately 1.2 km^2^(117 ha), whilst the village is approximately 0.2 km^2^(22 ha) in size [26]. The village has approximately 850 residences, located in a rough north–south pattern on the western side of the island, and is divided into 3 hamlets (Fig. 1). Average temperatures are relatively warmer and show much less seasonality in this area compared to North Queensland (Additional file 1: Figure S1).

Using a pre-existing but outdated basemap the entire village was surveyed and the map updated to reflect the current village structure of approximately 850 households. For each property, we recorded the name and contact details of the head of the household and the number of occupants. In addition, the number of small (<250 L) and large (>250 L) water containers present at each household was recorded. In total, approximately 2000 small and 2000 large containers were recorded within the village. Using this information, the village was divided into 47 zones, each zone having roughly the same number of large containers. As there were no street addresses, all properties were assigned a unique identifying code based on the zone in which it occurred and a number between 1 and 850.

To increase the likelihood of *Ae. aegypti* infected with *w*MelPop invading the population, a mosquito suppression campaign was undertaken. Local hamlet leaders were approached by project staff and asked to invite local community members to join the project team as paid project “collaborators”. These 47 collaborators worked with project staff and were responsible for the undertaking suppression, release and monitoring activities on the island.

Each collaborator was provided with a sweep net, and visited each house twice a week for 3 months prior to release, in an attempt to reduce larvae and pupae from each property. In addition, rubbish was removed and small containers turned upside down to reduce larval habitat.

Eggs for both release and back-up samples were hatched and reared in the IPNT insectary, where temperatures range between 26 and 31 °C. Larvae (400/bucket) were reared in 2 L buckets and fed a diet of Tetramin tropical fish pellets. When approximately 90 % of larvae had pupated, 20–40 larvae/pupae were placed into 900 individual 100 ml plastic cups for release. The cups were placed into wire racks, and stacked into polystyrene boxes for transport to the release site the following day to be released as pupae.

Quality of the release material was measured weekly by taking a random sample of larvae and adult females that were tested for *w*MelPop-CLA infection by PCR [24]. Egg viability was also monitored to determine the effectiveness of egg storage and incubation methods as well as transport between the release stock colony at NIHE (Hanoi) and the rearing facility at IPNT (Nha Trang). For each egg batch two subsamples of eggs were collected and counted at NIHE immediately after harvesting, and the number of viable eggs, dead eggs (collapsed) and hatched eggs recorded. One sample was kept at NIHE and the second sent with the other eggs to IPNT. Two days later the eggs were counted again and were hatched at both locations. In addition, pupae were retained in release cups to assess emergence and adult mortality after one week.

Releases were initiated on 3 April 2013 and consisted of pupae placed at every house. A small plastic basket was attached to the wall in an appropriate location (Additional file 1: Figure S2). These were generally away from the main living or sleeping areas of the house, high enough to avoid interference by children or pets and away from direct sunlight, wind and rain. Each week for 23 weeks, collaborators would place one cup of around 20 pupae into each basket (range 19–25) and mosquitoes were allowed to emerge. After 7 weeks of release the number of pupae per cup was increased to around 40 (range 37 – 51).

### Monitoring *Wolbachia* frequencies in Vietnam

For the purposes of the releases and monitoring, TN was divided into 47 zones in three hamlets (Fig. 1). To monitor the size of the mosquito populations and proportion with *w*MelPop infection, a network of 45 BG-S traps was established with monitoring starting from 27/12/12. As power supply on the island was unreliable, a battery backup system was utilized which allowed the traps to run for an additional 24 h should mains power fail. These were cleared weekly and mosquitoes sorted by species and sex. The *w*MelPop was detected in larval or adult samples by PCR using methods described above.

### Analysis

We followed the procedures outlined in Ritchie et al. [30] to estimate the size of populations of mosquitoes before release for the Australian releases where there was no suppression. Population estimates were obtained from counts of adult female *Ae. aegypti* from BG-S before release as compared to after the release. We compared changes in BG-S numbers with those obtained from a network of 12 BG-S traps placed outside the release sites around Parramatta Park in Cairns. The proportionate increase in BG-S counts 1 and 2 weeks either side of the release point was used to compute the population size before releases started by assuming different daily mortality rates (0.7-0.9) as considered in a previous study [30] for the release stock.

We followed changes in populations of mosquitoes by using BG-S numbers across time, and considered both the ovitrap data and BG-S frequencies to assess changes in *Wolbachia* frequencies following releases. We expected ovitrap data and BG-S trap data to match given that the latter were used after releases were terminated (except for the second period of male releases in MB). Binomial confidence intervals for the *Wolbachia* frequencies were computed assuming adults and larvae represented independent samples, although the larval estimates will underestimate variability because female *Ae. aegypti* generally deposit more than one egg in a container [ 27–29]. Correlation coefficients were computed to link BG-S catches to release numbers under different daily mortality rates.

For the TNI releases, it was not possible to estimate population size in this way because of suppression, a lack of evidence that BG-S numbers increased after the release period (see below), and strong temporal changes in mosquito population size known to occur at this site [26]. Nevertheless we did investigate the pattern of changes in BG-S numbers over time and compared estimates to the predicted number of infected females in the population from the releases as investigated previously, assuming different daily survival rates of 0.9 or 0.7 [30].

In past work, we have linked changes in *Wolbachia* frequencies and local mosquito numbers to different attributes of sites [29], showing that invasion rates depended on house attributes. We used a high density of ovitraps to determine these patterns but in the present releases data from a lower density of traps was available and only data from BG-S traps after the first few weeks. To test for local patterns within the release site, we therefore combined block data into 5 (BA) or 6 (MB) larger blocks (Fig. 1). These blocks differed in house characteristics. For BA, blocks varied in level of screening (from 59.1 % of residences in block 1 to 36.4 % in block 2 and 40 % in block 3). Block 1 also had a high proportion of brick houses (51.6 %) and houses positioned on the ground (46.0 %) compared to block 3 (23.2 % brick, 22.1 % to the ground). For MB, the percent screened houses varied from 59.2 % in block 2 and 56.8 % in block 1 to 34.2 % in block 4 and 37.3 % in block 5. Blocks 1 and 2 also had a high proportion of houses built at ground level (45.9 % and 58.2 % respectively) compared to blocks 4 and 5 (21.4 % and 17.3 % respectively). The latter house type consists of elevated “high-set” houses that often harbour large numbers of adult *Ae. aegypti* [31]. We used log likelihood ratios from contingency analyses to compare *Wolbachia* frequencies across blocks.

In TNI, there were no obvious features that distinguished houses in the different hamlets that defined the release area (Fig. 1c). Nevertheless we did test for any differences in *w*MelPop invasion rates across the three hamlets, by plotting data separately for each hamlet.

Although our main goal was to assess the ability of *w*MelPop to invade into an isolated area in comparison to the less virulent strain *w*Mel, we acknowledge that such a comparison is not directly possible because we are unable to release both strains at the same time in the same locations. However, we note that *w*Mel was able to rapidly invade other isolated areas near Machans Beach and Babinda (see reference [10]) when using similar release rates (as assessed by changes in population number) and when releases were stopped after *Wolbachia* frequencies in ovitraps reached a frequency of around 70 %. Moreover, we have been able to rapidly invade *w*Mel into the TNI, Babinda and Machans Beach in subsequent releases; these were terminated after a few weeks when frequencies reached 60-70 % based on BG-S monitoring but the infection then continued to increase to near-fixation and has been stable and high in Australian sites since that time.

## Results and discussion

### Deployment strategies for Australia and Vietnam

Two different approaches were utilised for release and community engagement in the two countries. In Australia *w*MelPop releases followed an approach used previously for *w*Mel [10]. Namely adult mosquitoes 3–7 days old were released from cups by our scientific team on a fixed grid every four houses without any prior suppression of the wild mosquito population. In Vietnam a team of local collaborating residents was used to assist with both pre-release suppression of wild mosquitoes as well as actual release of mosquitoes. While the pre-release suppression obtained by sweeping open containers reduced the larval population, there was no appreciable reduction in adult mosquito numbers. The suppression activities were however a valuable way to build a cohesive collaborator network whose residential status made them useful advocates for the acceptability of the project within the community. In addition a different life stage was released in Vietnam, namely pupae in small containers of water (Additional file 1: Figure S2). This was done to see if communities viewed this distribution modality more acceptable than adult releases. However responses from the community suggest that this was not the case and that adult releases appear to be less obtrusive and more acceptable. Both release strategies resulted in increases in *w*MelPop frequencies to high levels (see below).

### Population size estimates

**Fig. 2:**
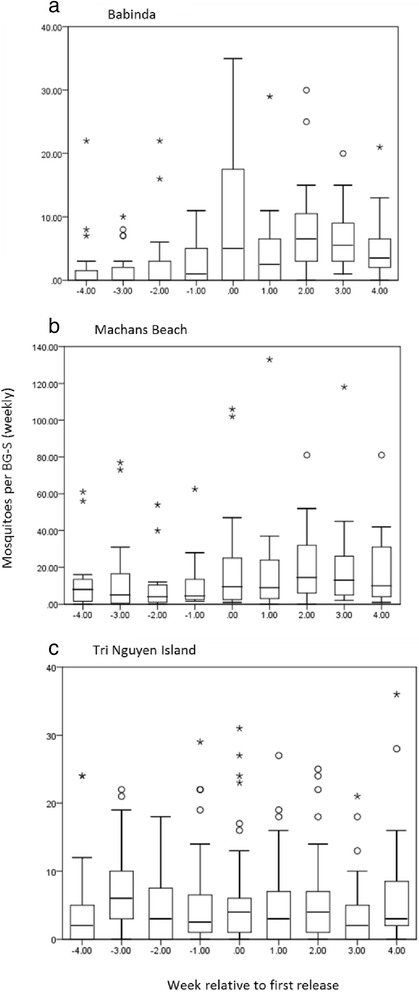
Box plots for BG-S counts at (**a**) Babinda, (**b**) Machans Beach and (**c**) Tri Nguyen. Numbers on x axes indicate week of first release (0), and before/after first release. Symbols on the plots represent extreme outliers

For the Australian sites, there was an increase in sampled adult numbers directly after releases were initiated (Fig. 2a,b). The changes in abundance of mosquitoes before and during the release in Australia were used to estimate population size under different estimates of daily survival (Table 1) following Ritchie *et al.* [30]. Estimates based on a mortality rate of 0.8 suggest low numbers of adult females (2–5) per premise in Babinda (BA) compared to a much higher number (8–17) at Machans Beach (MB). Based on a 0.8 daily mortality rate, we therefore estimated that the total population size of uninfected adult mosquitoes at the site before release was around 2500 for BA and 10000 for MB. However it should be emphasized that confidence intervals around these estimates are large (Table 1), translating into an estimated range of 1400–3700 for Babinda and 6500–13000 for Machans Beach.

**Table 1:** Estimated number of wild female *Ae. aegypti*/ daily survival in Babinda and Machans Beach

Locale	Parameter	Adult daily survival		
		0.7	0.8	0.9
Babinda	Mean BGS collection before, after release		0.32, 0.95	
	Expected no./ premise for DS = 0.7, 0.8 and 0.9	4.54	6.52	11.12
	Estimated no. collected/BGS/day	0.95	0.95	0.95
	Wild female *Ae. aegypti*/premise	2.25	3.24	5.52
	95 % CI	1.2–3.3	1.8–4.7	3.0–8.0
Machans Beach	Mean BGS collection before, after release		0.86, 1.75	
	Expected no./ premise for DS = 0.7, 0.8 and 0.9	9.08	13.24	22.58
	Estimated no. collected/BGS/day	0.89	0.89	0.89
	Wild female *Ae. aegypti*/premise	8.77	12.80	21.82
	95 % CI	5.8–11.8	8.4–17.1	14.4–29.2

Estimated number of wild female *Ae. aegypti*/premise based on three levels of daily survival in Babinda and Machans Beach. Wild female *Ae. aegypti* populations estimated from estimated number of released mosquitoes and relative increase change in BG-S collection as the recapture rate. Confidence intervals of the estimated female population were calculated by multiplying the estimated population by the CIs (as a proportion of the mean) for the BGS collection from 2 weeks before release

**Fig. 3:**
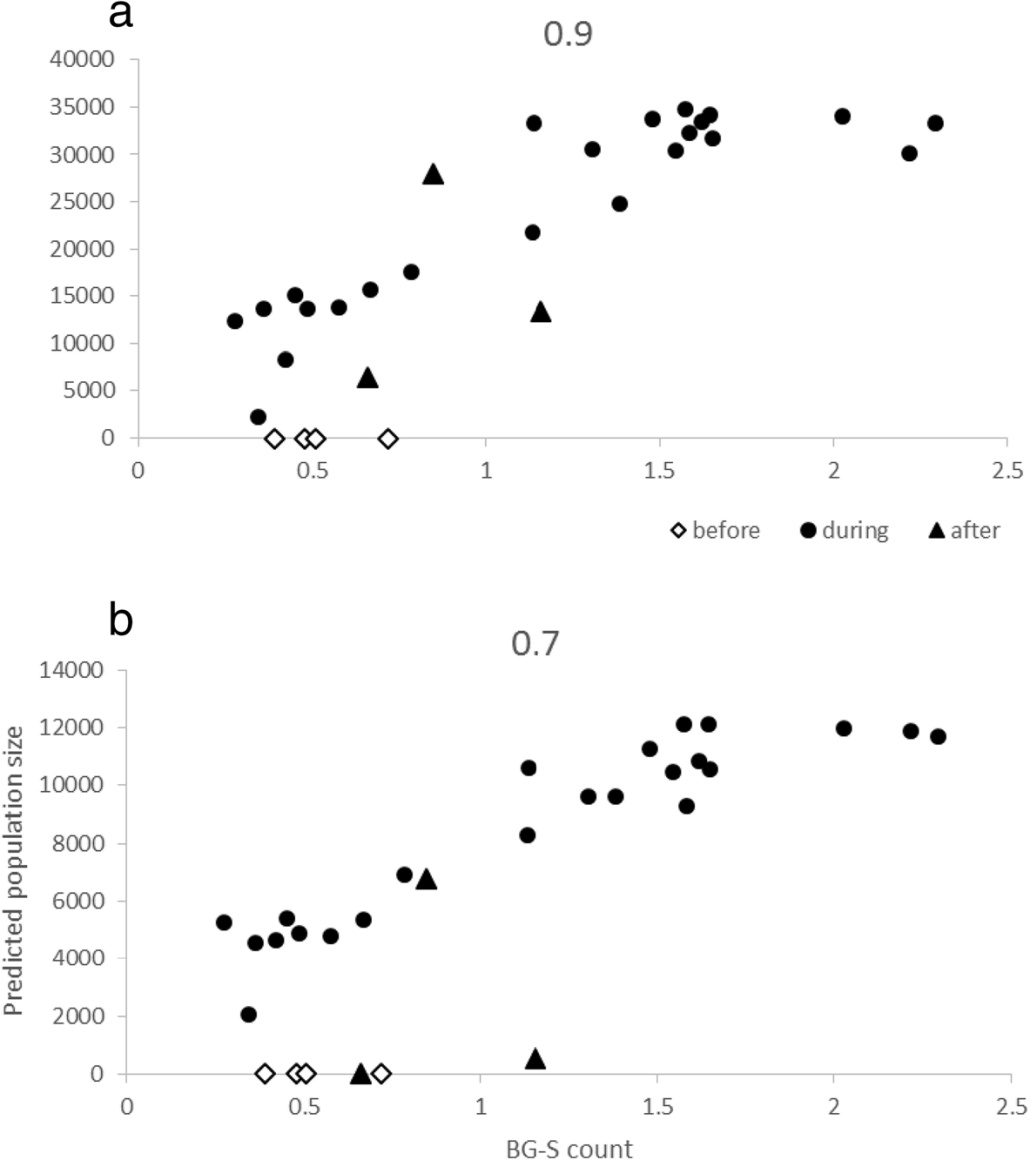
Observed density of females in the Tri Nguyen population during the release from BG-S counts (x axis) plotted against predicted numbers of release individuals present in the population (y axis) based on two values of daily mortality, (**a**) 0.9 and (**b**) 0.7. BG-S counts before the first release and predicted numbers after releases finished (but when released individuals were still expected to be present in the population) are also presented

In Vietnam there was no evidence of an increase in adult counts after releases started (Fig. 2c), unlike in the Australian sites and despite population suppression efforts. BG-S counts did increase after a few weeks, and when these are plotted against the estimated number of females in the population derived from release material with different estimates of daily mortality, there was a strong positive association (Fig. 3) both when daily mortality was assumed to be 0.9 (r = 0.89, N = 23, P < 0.001) and 0.7 (r = 0.93, N = 23, P < 0.001). These graphs also highlight the lack of an increase in BG-S counts after releases were started and the sharp drop in BG-S counts soon after releases were terminated.

### Frequency changes in Babinda

**Fig. 4:**
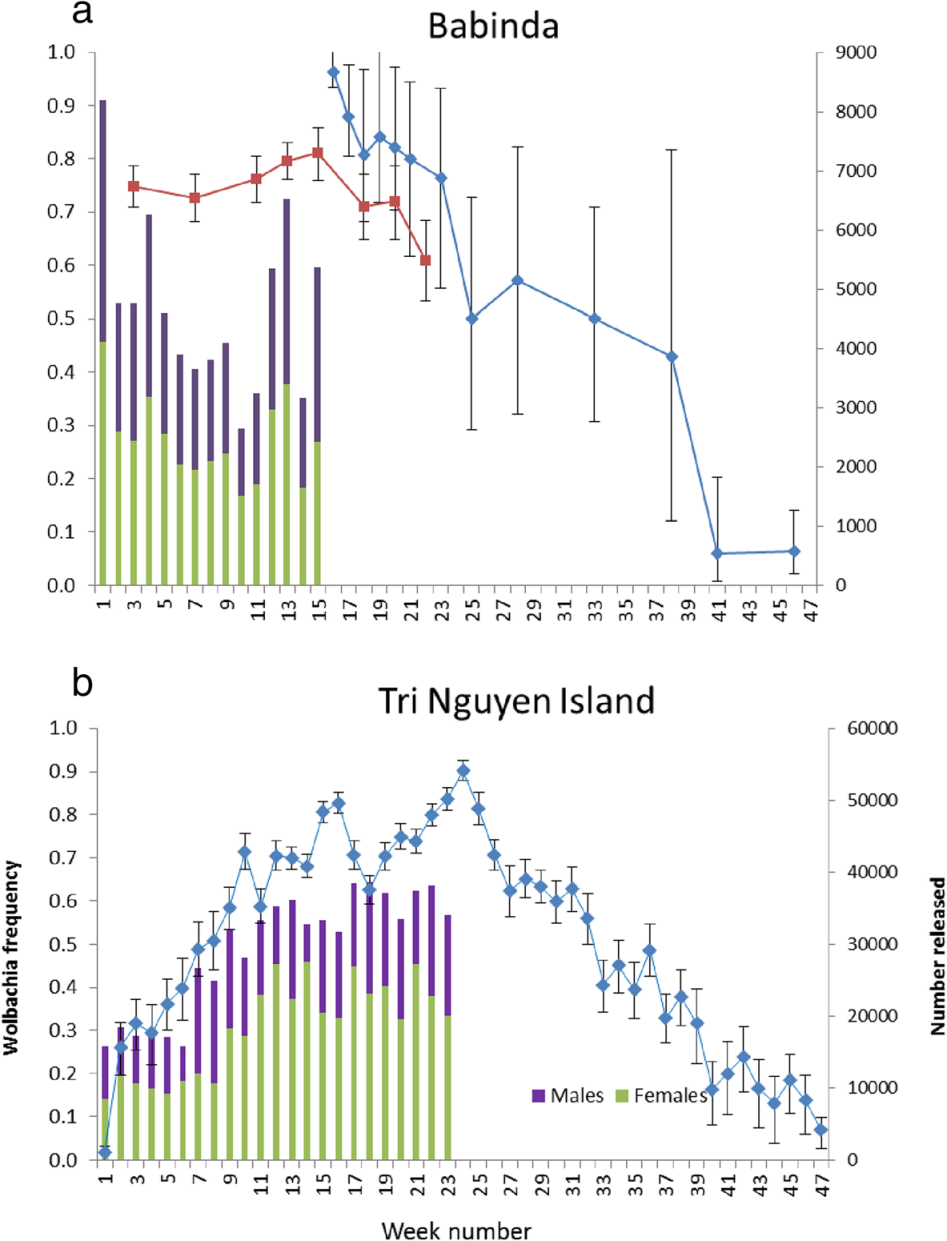
Changes in frequency of the infection and release numbers at (**a**) Babinda and (**b**) Tri Nguyen. For Babinda frequencies estimated from ovitraps are given in red, and those from the BG-S traps are given in blue. Release numbers are plotted separately for the two sexes. Error bars represent binomial 95 % confidence intervals

The frequency of the infection at Babinda rapidly increased following initiation of releases to around 70-90 %, as initially detected through ovitraps and later through the BG-S traps (Fig. 4a). However the infection failed to reach frequencies near fixation despite ongoing releases and in contrast to the pattern seen for *w*Mel in nearby Gordonvale following releases in 2011 [10]. Following termination of the releases in April, 15 weeks after releases started, there was a decrease in infection frequency which dropped to less than 10 % after 41 weeks since the start of releases (Fig. 4a).

**Fig. 5:**
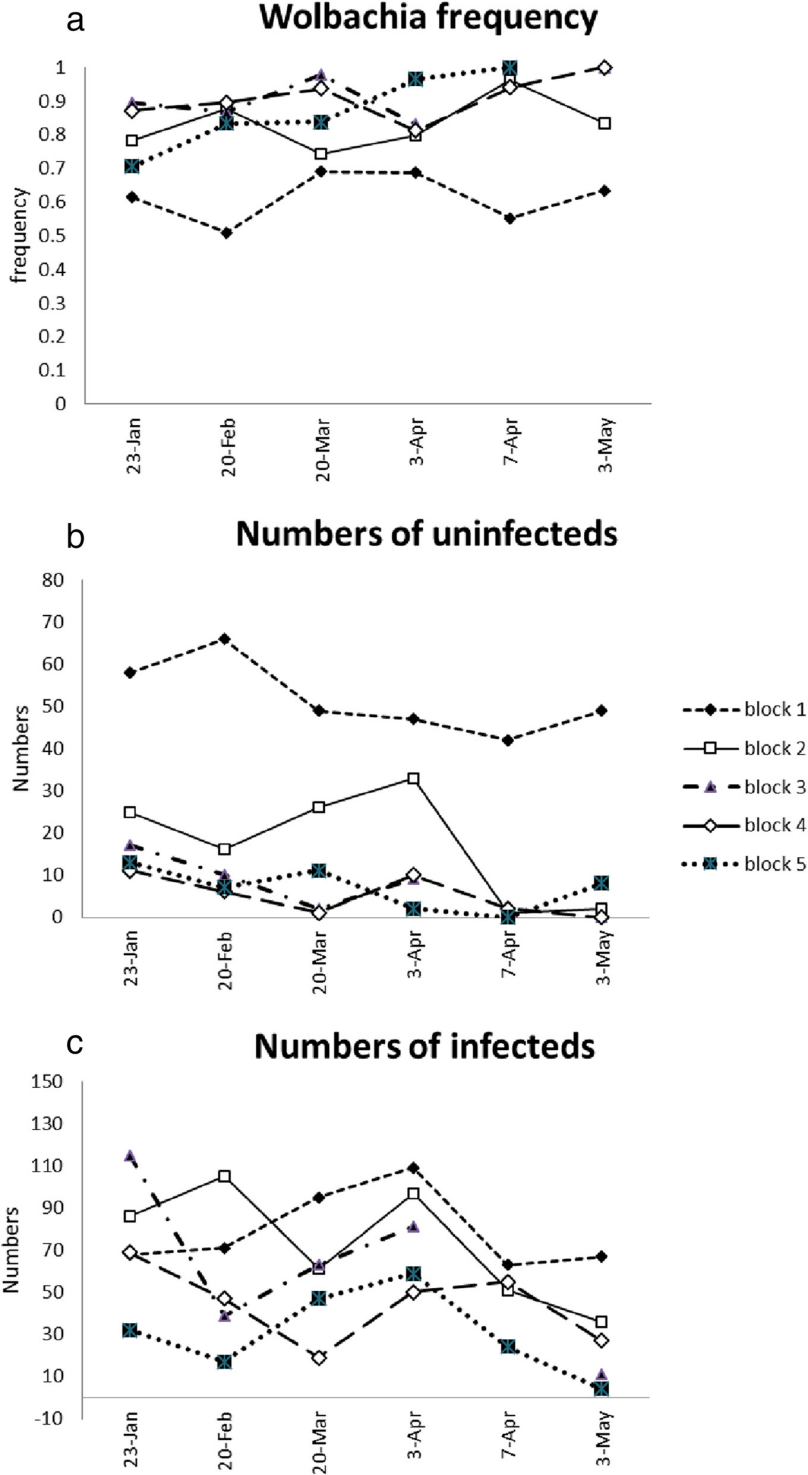
Changes in (**a**) *Wolbachia* frequency, (**b**) number of uninfected individuals and (**c**) number of infected individuals in Babinda blocks as assessed by ovitraps. The *Wolbachia* frequencies as well as numbers of uninfected and infected larvae detected are given

The initial invasion at Babinda as monitored in the ovitraps was not uniform across the release area. This was evident from trap data accumulated across the blocks designated in Fig. 1 and plotted separately for these blocks in Fig. 5. A contingency analysis indicated a significant difference in the incidence of *Wolbachia* infected mosquitoes across all six collections (likelihood ratio, G, ranging from 28.69 to 54.66, df = 4, *P* < 0.001 in all cases). An area covered by block 1 showed a relatively lower frequency of the infection during the entire release period, which remained around 60 %. In this area the number of uninfected mosquitoes was relatively high based on ovitrap data (Fig. 5b) whereas infected egg numbers also tended to be high but much more similar to numbers seen in the other blocks (Fig. 5c). *Wolbachia* frequencies were also lower in block 2 where the number of uninfected mosquitoes tended to be relatively high.

### Frequency changes in Machans Beach

**Fig. 6:**
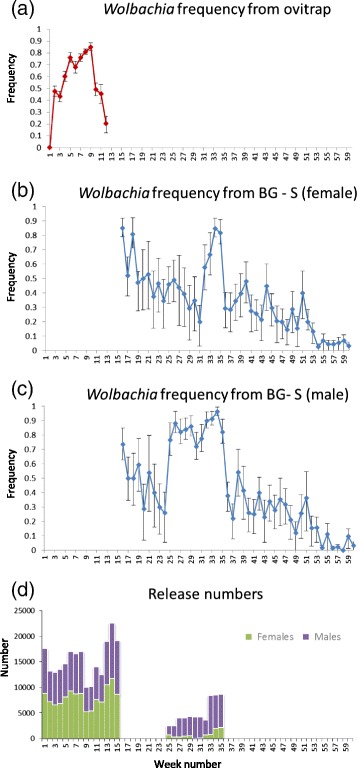
Changes in frequency of the infection and release rates at Machans Beach. *Wolbachia* frequencies from ovitraps and BG-S traps are plotted separately for the two sexes along with release numbers for the two sexes. Error bars represent binomial 95 % confidence intervals

Releases led to a slower increase in infection frequencies at Machans Beach compared to Babinda, moving to 0.4-0.5 initially (Fig. 6) rather than to 0.7 as at Babinda and despite the higher release rate at this site. Nevertheless the infection frequency did exceed 0.8 by week 17 in April when releases were terminated, with a similar high infection recorded in both the ovitrap and BG-S collections (Fig. 6a, b, c). This was followed by a decrease in infection frequency to around 0.3, again with consistent patterns for the two trapping methods, and in contrast to the pattern seen for *w*Mel in nearby Yorkeys Knob where the infection continued to increase to near fixation from such a frequency [10]. The decrease of *w*MelpPop suggests that much of the infected population collected in the traps may have consisted of released mosquitoes. This was reiterated when subsequent male releases resulted in an increase in frequency again to a high estimate exceeding 0.9 by week 33, at which stage releases were terminated, and frequencies again dropped (Fig. 6b, c). Infection frequencies detected in the BG-S traps were higher for males than females as expected because traps would have collected males from the releases [32]. There was a subsequent decrease in infection frequency to around 0.4, at which stage the frequency decreased relatively slowly across several weeks extending into the early part of 2013 at week 53 (Fig. 6).

As was the case at Babinda, the initial increase in infection frequency varied across the area as documented by the ovitraps (Additional file 1: Figure S3a), occurring slowly in block 5 and block 4 along the shoreline, and much more rapidly in block 1, with significant differences in infection frequencies across blocks by contingency tests in the period to 11 April (with G values ranging from 68.41 to 14.11, df = 4, all *P* values < 0.10). These differences are likely to partly reflect variation in the abundance of uninfected mosquitoes in the blocks at the start of the release; numbers of uninfecteds were particularly low in block 1 (Additional file 1: Figure S3b), likely increasing the rate of invasion (Additional file 1: Figure S3c).

There was also spatial variability in the loss of the infection in the different blocks in September, with a very rapid loss in block 4 compared to a much slower loss in block 2 where the infection persisted at intermediate frequencies for several weeks (Additional file 1: Figure S3d). Infection frequencies differed significantly between blocks on 5 of the 7 occasions in the July-September period plotted in Additional file 1: Figure S3d) by contingency tests (with G values ranging from 7.01 (df =4, *P* = 0.17) to 35.03 (df = 4, *P* < 0.001)). These different loss rates coincided with numbers of mosquitoes in the traps; block 4 had consistently high numbers of mosquitoes in this period, while numbers in block 2 were low (Additional file 1: Figure S3e). Unlike at the start of the release period, mosquito numbers in block 5 were not particularly high relative to the other blocks.

### Frequency changes in Tri Nguyen

At Tri Nguyen, Hon Mieu Island (TNI), the *Wolbachia* frequency in adults from BG-S traps increased rapidly to around 30 % in the first 3–5 weeks, and then increased again to around 70 % in weeks 10–20, reaching nearly 90 % at the end of the release period (Fig. 4b). Once releases were terminated after 23 weeks, there was a decline over a period of 20 weeks and the *Wolbachia* frequency was <20 % by this time. The increase in infection frequency was similar in all three hamlets (contingency tests, all *P* values > 0.05), as was the number of uninfected mosquitoes (Additional file 1: Figure S4a, b), although the number of infected and uninfected mosquitoes tended to be lower in Hamlet 3 at the end of the release period compared to the other hamlets (Additional file 1: Figure S4b,c).

In these releases the *w*MelPop infection failed to become sustainably established in two populations of *Ae. aegypti* in north eastern Australia and in one island release site in Vietnam. This contrasts with the recent and ongoing success of releases with *w*Mel infected *Ae. aegypti*, which has led to stably infected populations across a 3- year period [12]. The *w*Mel releases succeeded despite a relatively short 10-week release period [10], whereas the *w*MelPop infection failed to permanently establish despite a longer release period, particularly at TNI where releases were carried out across 23 weeks. Moreover, the difficulty of establishing *w*MelPop in contrast to *w*Mel has further become evident from subsequent releases of *w*Mel in Babinda and Machans Beach; in these subsequent releases, the *w*Mel infection has continued to increase from a frequency of 60–70 % (assessed via BG-S traps) when releases were terminated to become stably established at a high frequency at all sites (Eliminate Dengue, unpublished results).

Based on fitness tests in the laboratory and field, we expected invasion of *w*MelPop to be challenging, particularly in the dry season. The *w*Mel infection has a fecundity cost of around 15 %, whereas *w*MelPop has a larger fecundity cost as well as other potential costs connected to lifespan reduction [6, 19], feeding and probing behaviour [18, 33], activity [15] and the location of field resources [32]. In addition, there is a substantial cost associated with quiescent eggs [7, 19]; *w*MelPop infected eggs when in a dried state tend to lose viability over time, in contrast to uninfected eggs or *w*Mel infected eggs that only experience a small reduction in viability even across several months. Recent experiments also point to a fitness cost associated with an extended larval period when larvae are cultured at a high density [17], which was avoided in the low density rearing environment used for generating mosquitoes for release. The additional fitness costs associated with *w*MelPop likely reflect the higher density of *Wolbachia* in body tissues. These fitness differences among *Wolbachia* strains have made it more challenging to get invasion of *w*MelPop into uninfected populations of *Ae. aegypti* in semi-field cages when compared to *w*Mel [5].

When the deleterious fitness effects of *w*MelPop are taken into account, it is perhaps unsurprising that the *Wolbachia* failed to establish in the natural populations, unlike in the semi-natural field cages trialled earlier [5]. Although we were able to increase *w*MelPop frequencies in larvae and adults to high levels for a substantial period, this was nevertheless insufficient for sustained establishment. Previous calculations have suggested that the unstable equilibrium point needed for invasion by this infection can be high under dry conditions when aestivating eggs are maintained in a dried state; because of this factor, the unstable point may be >80 %, compared to around 40 % in the wet season [19], although there was ongoing rainfall after releases were terminated in both Babinda and TNI (Additional file 1: Figure S1). These calculations are based on the assumptions of a population being closed and with maternal transmission being complete. Maternal transmission of *w*Mel under field conditions in isolated populations near Cairns appears to be very high, and a small percentage of individuals in these populations seem to consist of immigrants [12]. Maternal transmission and movement rates have not been tested in Tri Nguyen although a few *Wolbachia*-infected mosquitoes have been detected at BG-S traps placed at the port from which boats leave to travel to the island, suggesting the population is not entirely closed.

The *w*MelPop infection in TNI failed to establish despite attempts to suppress the natural population with source reduction prior to releases being initiated. While the number of uninfected adults in the population remained relatively constant during the first few weeks of the release, the number of infected mosquitoes caught in BG-S traps increased as release numbers were ramped up. The reasons for the lack of an initial increase in adult numbers in BG-S traps soon after releases started are unclear. While releases in Australia involved adults whereas those in TNI involved pupae, we did not expect a large difference in emergence times due to this factor because pupae at TNI emerged within a day. However, mosquitoes released as pupae were not sugar-fed or mated at the time of release, and were also vulnerable to domestic control methods and predation by geckos. The lack of an increase in adult numbers may be related to a high level of adult mortality initially at TNI due to dry conditions that immediately preceded the release, although it is unlikely that there would have been a high level of egg mortality [34]. Any effects of movement will be exacerbated if *w*MelPop causes a reduction in population size in the dry season [8], potentially providing vacant breeding sites for any uninfected individuals entering the population.

These releases suggest that it will be difficult to get *w*MelPop established into populations unless the populations are completely isolated, wild populations have a relatively low density and potential fitness costs of *w*MelPop are as low as possible due to permissive environmental conditions. Invasion and persistence may be more likely in areas where the density of mosquito larvae is low and breeding containers are regularly inundated with water to limit population replenishment from quiescent eggs. Invasions by *w*MelPop may also be facilitated by reductions in populations of adults and immature stages before a release occurs [35, 36]. However these strategies were clearly inadequate on TNI and will be challenging to implement in populations that are not isolated. Nevertheless, *w*MelPop when introduced into *Ae. aegypti* has desirable qualities including strong blocking of arboviruses [3, 14] and also the potential to reduce the size of mosquito populations when populations experience a marked dry season and might be useful to produce suppression in areas such as southern Queensland as long as the infection was able to invade populations [8].

The ovitrap and BG-S data provided similar patterns of frequency changes of *Wolbachia* at both locations where data were available at the same time (towards the end of the release period and immediately after release). However, BG-S frequencies were initially higher at Babinda than comparable ovitrap frequencies (Fig. 2), no doubt reflecting the fact that many of the individuals caught in the BG-S traps would have been females from the released population that may not yet have fed and matured. A similar pattern was evident at Machans Beach (Fig. 3). These adults are on average substantially larger than adults from natural breeding sites and also differ in wing shape so it is possible to separate most of them based on these characteristics [32]. Ovitraps provide a better way of monitoring invasions during the release period but are more time consuming because traps need to be placed out and retrieved on each occasion, and BG-S traps provide a convenient approach for ongoing monitoring after releases are completed.

Both the invasion and loss of the *w*MelPop infection was heterogeneous across the target site. Previous analyses have shown that in Gordonvale, one of the *w*Mel release areas, local invasion rates depended on mosquito density and house attributes; high densities of uninfected mosquitoes occurred where houses tended to be open and wooden, and slowed invasion rates [29]. In the current releases, these factors may explain the slow rate of invasion in two of the blocks in Machans Beach where houses tended to be open and where they may have harboured higher mosquito populations. In addition, the infection in Machans Beach persisted in an area where mosquito numbers were relatively low. Perhaps there was an abundance of small breeding sites in this area with a rapid turnover of eggs, reducing deleterious effects linked to *w*MelPop. The reasons for the heterogeneous invasion of Babinda are unclear; where invasion proved difficult, houses tended to be low to the ground and screened. Regardless, these patterns point to challenges in introducing *w*MelPop across an area.

## Conclusions

While *w*MelPop did not persistently establish in any of the three sites, it was possible for high frequencies of *Wolbachia* to be generated in the adult population (>80 %) during active releases when there would have been a mix of release mosquitoes and those emerging from natural breeding sites. Given the very strong dengue blocking properties of this *Wolbachia* strain, this level of establishment may influence dengue transmission in release areas [13]. The lack of invasion was most likely due to deleterious fitness effects associated with this *Wolbachia* infection and presumed migration of uninfected mosquitoes into populations. For *w*MelPop to sustainably establish, other strategies would be required such as developing an association between pesticide resistance and the *Wolbachia* infection [37]. In additional releases in all of these locations with the *w*Mel infection, *Wolbachia* has now successfully invaded despite a much shorter release period and BG-S trap frequencies that were lower than those observed here (unpublished data), emphasizing the fact that *w*Mel, unlike *w*MelPop, can readily invade local areas under a range of conditions.

## Additional file

Additional file 1: Figure S1. Weekly rainfall and average temperature at the three release sites during the release period (marked by a blue bar) and in the ensuing weeks. Climate data for Machans Beach came from the nearby Cairns airport station (Bureau of Metereology Australia). For Tri Nguyen, temperature data came from Nha Trang while rainfall data was collected from the island. **Figure S2.** Release basket used in Vietnam for pupal releases. **Figure S3.** Changes in *Wolbachia* frequency and catch numbers at the block level at Machans Beach. **Figure S4.** Changes in *Wolbachia* frequency and mosquito numbers at Tri Nguyen. (DOCX 1283 kb)
