# Supplementary figures and images for: Field evaluation of the establishment potential of wmelpop Wolbachia in Australia and Vietnam for dengue control
Source: Parasit Vectors. 2015 Oct 28;8:563. doi: 10.1186/s13071-015-1174-x (PMC4625535; doi:10.1186/s13071-015-1174-x)

**
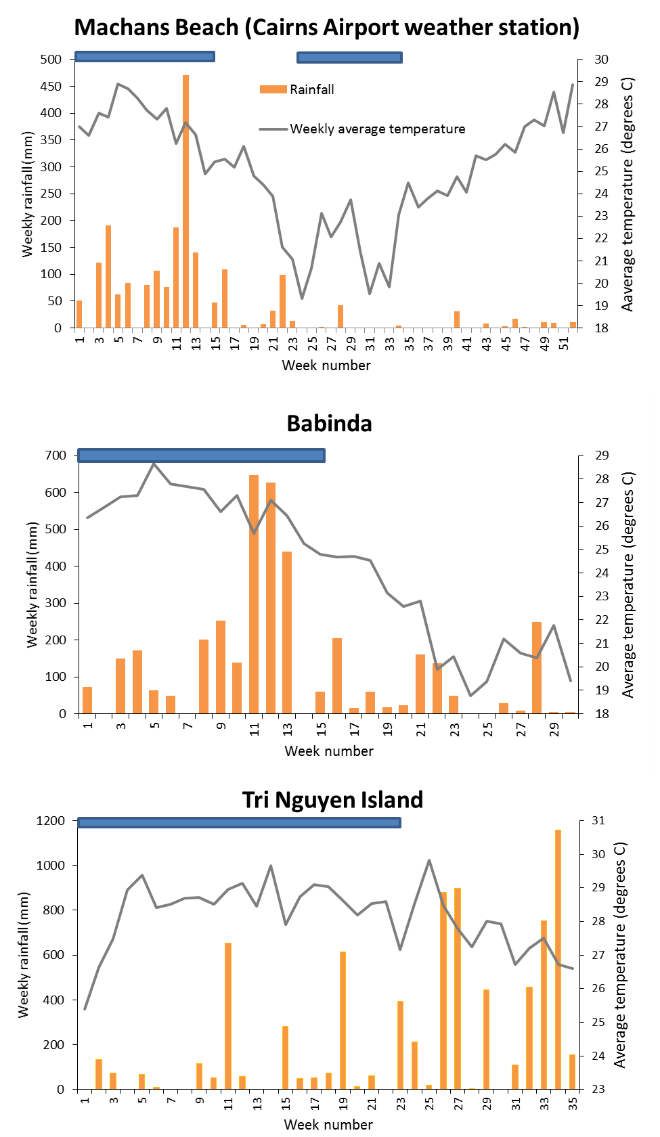
Figure S1.**

**Figure S2.**


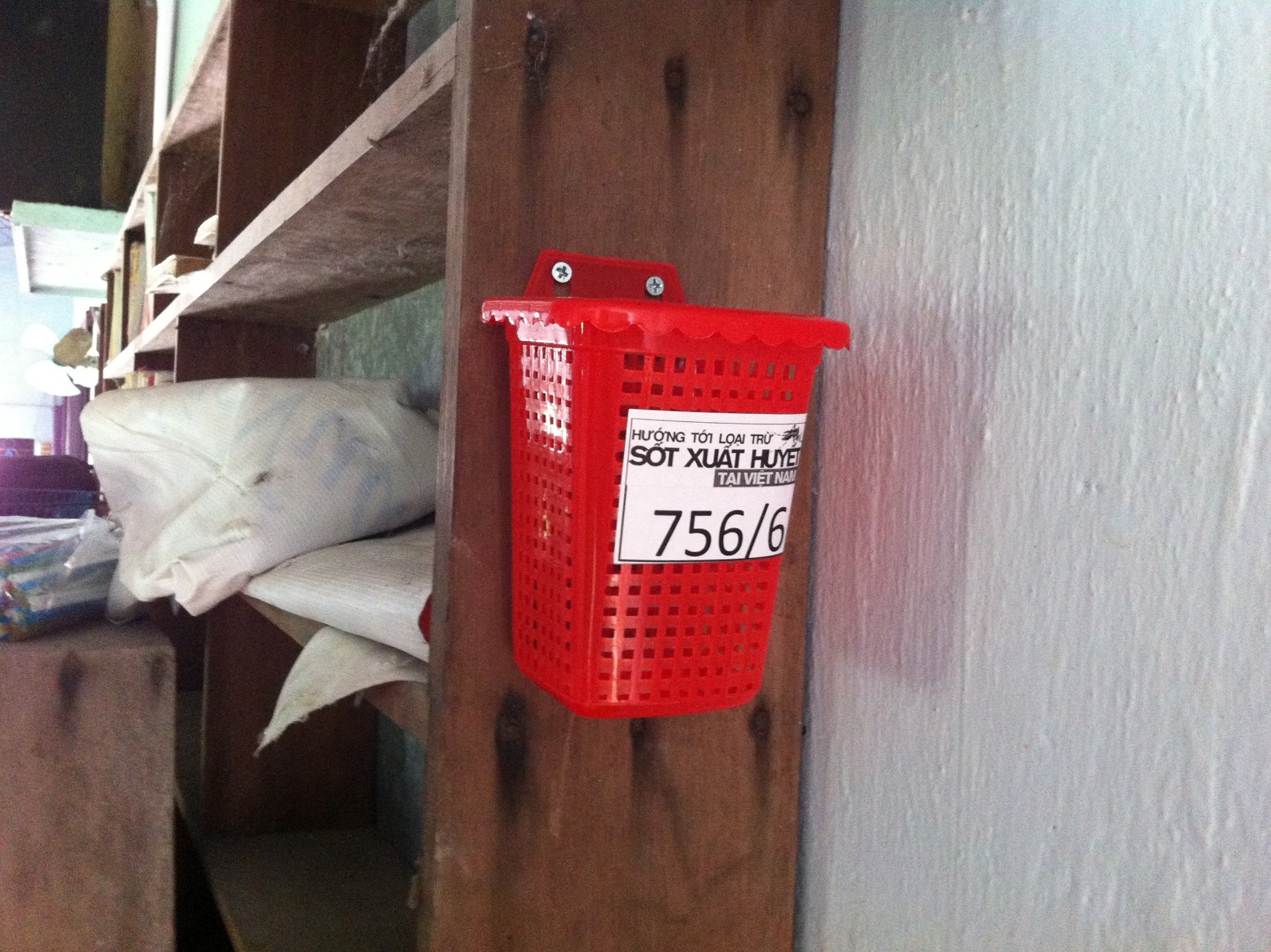


**Figure S3.**


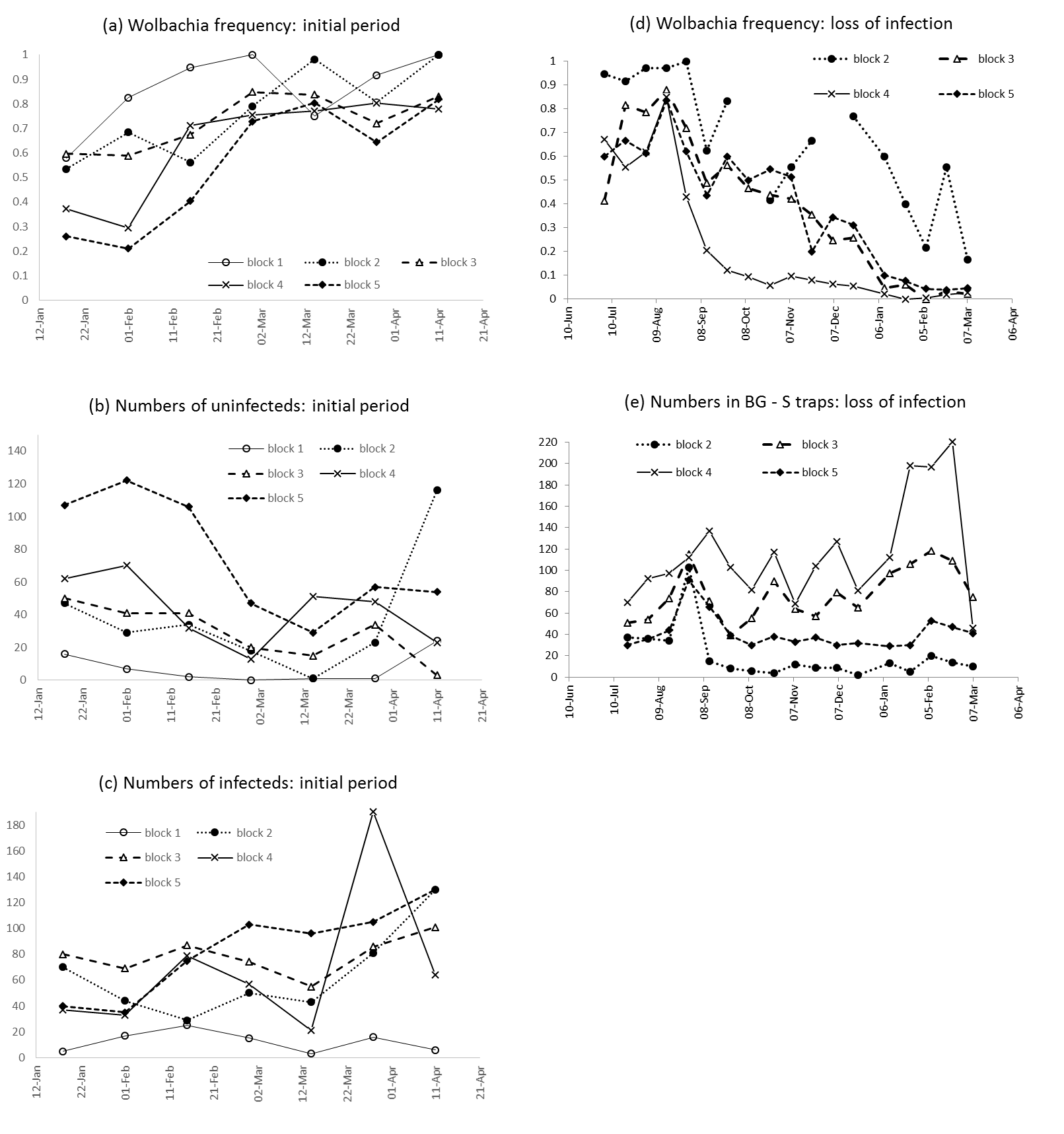


**Figure S4.**


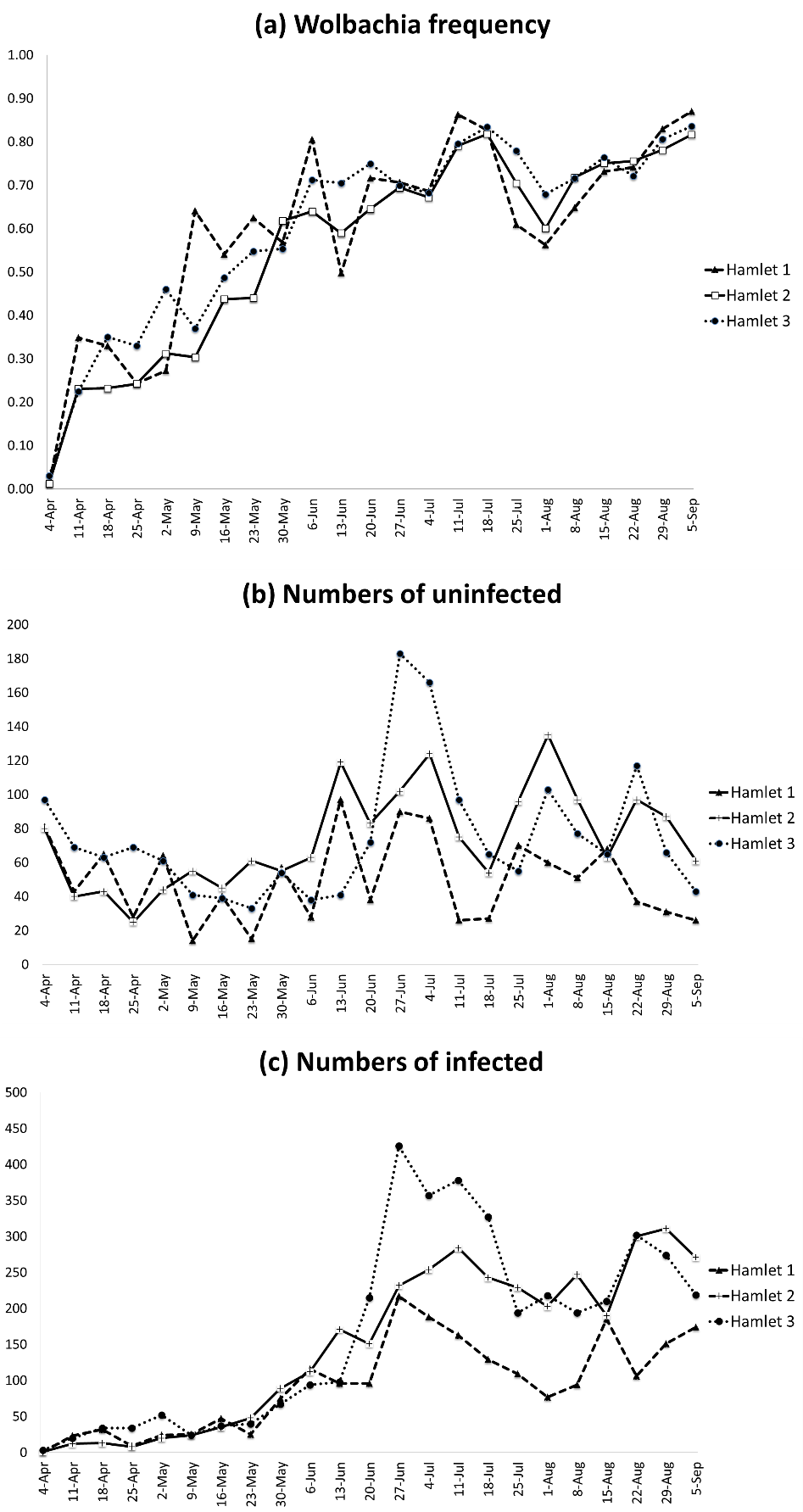

Supplement: Additional file 1: Figure S1. — Weekly rainfall and average temperature at the three release sites during the release period (marked by a blue bar) and in the ensuing weeks. Climate data for Machans Beach came from the nearby Cairns airport station (Bureau of Metereology Australia). For Tri Nguyen, temperature data came from Nha Trang while rainfall data was collected from the island. Figure S2. Release basket used in Vietnam for pupal releases. Figure S3. Changes in Wolbachia frequency and catch numbers at the block level at Machans Beach. Figure S4. Changes in Wolbachia frequency and mosquito numbers at Tri Nguyen. (DOCX 1283 kb) [file 13071_2015_1174_MOESM1_ESM.docx]
